# AMTDB: A comprehensive database of autophagic modulators for anti-tumor drug discovery

**DOI:** 10.3389/fphar.2022.956501

**Published:** 2022-08-09

**Authors:** Jiahui Fu, Lifeng Wu, Gaoyong Hu, Qiqi Shi, Ruodi Wang, Lingjuan Zhu, Haiyang Yu, Leilei Fu

**Affiliations:** ^1^ School of Traditional Chinese Materia Medica, Key Laboratory of Structure-Based Drug Design and Discovery, Ministry of Education, Shenyang Pharmaceutical University, Shenyang, China; ^2^ Sichuan Engineering Research Center for Biomimetic Synthesis of Natural Drugs, School of Life Science and Engineering, Southwest Jiaotong University, Chengdu, China; ^3^ State Key Laboratory of Biotherapy and Cancer Center, West China Hospital, Sichuan University, Chengdu, China; ^4^ State Key Laboratory of Component-based Chinese Medicine, Tianjin University of Traditional Chinese Medicine, Tianjin, China

**Keywords:** AMTDB, database, autophagy, autophagic modulator, anti-tumor drug

## Abstract

Autophagy, originally described as a mechanism for intracellular waste disposal and recovery, has been becoming a crucial biological process closely related to many types of human tumors, including breast cancer, osteosarcoma, glioma, etc., suggesting that intervention of autophagy is a promising therapeutic strategy for cancer drug development. Therefore, a high-quality database is crucial for unraveling the complicated relationship between autophagy and human cancers, elucidating the crosstalk between the key autophagic pathways, and autophagic modulators with their remarkable antitumor activities. To achieve this goal, a comprehensive database of autophagic modulators (AMTDB) was developed. AMTDB focuses on 153 cancer types, 1,153 autophagic regulators, 860 targets, and 2,046 mechanisms/signaling pathways. In addition, a variety of classification methods, advanced retrieval, and target prediction functions are provided exclusively to cater to the different demands of users. Collectively, AMTDB is expected to serve as a powerful online resource to provide a new clue for the discovery of more candidate cancer drugs.

## Introduction

Autophagy is the process by which cells clean up abnormal and redundant substances, such as proteins, nucleic acids, and damaged organelles, and produce new substances for reuse by the body. Normally, autophagy is maintained within a reasonable range, which is beneficial to keep human cells healthy, resist aging and resist the invasion of diseases (Aman et al., 2021). Nonetheless, abnormal autophagy can have a significant impact on the occurrence and development of diseases, which are fully unraveled by tumor diseases. On the one hand, autophagy removes impaired mitochondria and attenuates the production of reactive oxygen species (ROS), thereby avoiding the accumulation of DNA damage, which may induce tumorigenesis and development and increase susceptibility to cancer. (Filomeni et al., 2015). In addition, activated autophagy induces programmed cell death and restrains tumor cell growth (Meyer et al., 2021). Similarly, autophagy together with ubiquitinated proteins can mediate the clearance of p62, an adaptor protein that binds microtubule-associated protein 1 light chain 3 (LC3) to ubiquitin on ubiquitin and is upregulated in cancer cells, and induction of autophagy can alleviate p62 accumulation and inhibit tumorigenesis (Mathew et al., 2009). On the other hand, autophagy provides sufficient energy and nutrients for the rapid division of cancer cells, especially in areas of low perfusion where nutrients and oxygen are limited. Activated autophagy protects tumor cells from the control of therapeutic drugs or increases drug resistance to interfere with the therapeutic effect (Zhang et al., 2015; Zhuang et al., 2022). Therefore, manipulating autophagy with compounds is a promising approach to tumor intervention, which plays the purpose of treating tumors by inhibiting autophagy and inducing autophagy to prevent the occurrence of tumors.

Recently, increasingly autophagy regulators have been reported, such as BL-918, NVP-BEZ235, perifosine, 3-methyladenine (3-MA), and so on. In particular, the successful use of chloroquine (CQ) and hydroxychloroquine (HCQ) in clinical practice have greatly stimulated the design and development of autophagy regulators (Fu et al., 2022). Protein kinases like adenosine 5-monophosphate activated protein kinase (AMPK), AKT, unc-51-like kinase 1 (ULK1), transcription factors such as transcription factor EB (TFEB), and hypoxia-inducible factors (HIFs), other “druggable” targets as Dopamine receptors, adrenergic receptors, etc. are all regulatory targets of autophagy compounds (Zhang et al., 2017; Li et al., 2021; Zhang et al., 2021). Herein, it is very important to establish a comprehensive information platform to collect the above information.

Although some online resources, including ACDB, HAMdb, and PubMed, have provided chemical information on autophagy regulators and autophagy-related genes, these databases only contain partial information and have not been effectively organized. The accumulating deposited autophagy compounds and targets, the development of antitumor drugs, and the elucidation of the relationship between tumors and autophagy all urgently require the emergence of a new database. In such cases, we focused on compounds and targets, and constructed a comprehensive database of autophagy modulators (AMTDB), providing users with a high-quality and practical online platform. Information such as International Union of Pure and Applied Chemistry (IUPAC) Name, CAS, tumor type, and molecular mechanism are all covered comprehensively. Importantly, the AMTDB database also provides target prediction services, to provide potential targets for the discovery of autophagy regulators, paving a creditable avenue for the development of anti-tumor drugs.

## Materials and methods

### Data collection and curation

**FIGURE 1:**
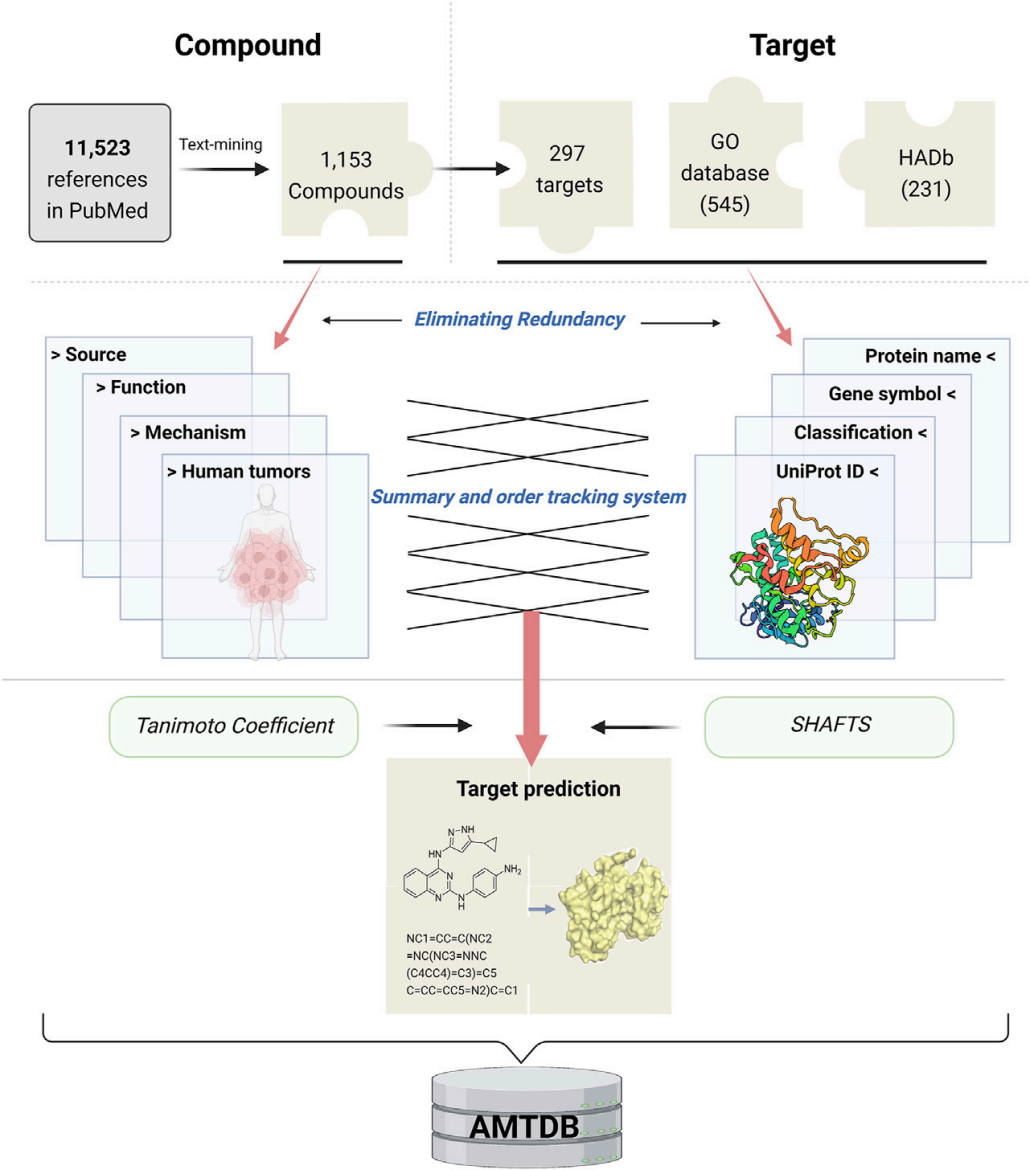
A logical framework of AMTDB.

AMTDB is a comprehensive pool of compounds and proteins, and its total data collection and curation process is shown in Figure 1. Using “ [autophagy (Title/Abstract)] AND {[inhibitor (Title/Abstract) OR activator (Title/Abstract) OR agonist (Title/Abstract) OR antagonist (Title/Abstract)]}” as the search term, we performed text mining on 11,523 articles, collected from PubMed, including research articles and reviews, and manually typed in 1,153 autophagy regulators and 297 targets. Of course, in order to ensure the reliability of the data, a third person was allowed to verify the original data set. Notably, articles that did not introduce autophagy compounds were considered negatives, and repeated data were considered false positives, which were not recognized and included. Finally, after the above data processing is completed, a simple random sampling method is used to confirm the final data. The data number is obtained by the function “= RAND ()” in EXCEL. For these modulators, we have retained and supplemented information such as compound structure, SMILES, IUPAC Name, CAS numbers, and references. At the same time, we also summarized its effect on autophagy (e.g., activating/inhibiting), molecular mechanism, and tumor content. Importantly, the regulatory targets of compounds were the focus of our emphasis.

Two approaches were used to gather gene and protein information. One is based on the autophagy-related genes provided by the existing Gene Ontology (GO) and HADb databases, and the two data collections were summarized and eliminated to obtain 660 genes in total. Another way is to add new data from the compound targets that have been sorted out (i.e., reported in the literature). All the above gene/protein information was cleaned again with the ID provided by UniProt, and 860 key information was finally obtained. Notably, given that the database is intended to assist in the design and discovery of new anti-tumor drugs, here the UniProt ID corresponds to *Homo sapiens*, and homology mapping is required if other species are to be studied.

The above data are collected and collated using multiple databases, as shown below:

Chemical Book (https://www.chemicalbook.com/)

GO database (http://geneontology.org/)

HADb (http://www.autophagy.lu/)

MCE (https://www.medchemexpress.cn/)

PubChem (https://pubchem.ncbi.nlm.nih.gov/)

PubMed (https://pubmed.ncbi.nlm.nih.gov/)

UniProt (https://www.uniprot.org/)

Web of Science (http://www.webofscience.com/)

SciFinder (https://scifinder.cas.org/)

### Classification principles for data

To manage and account for the above data more clearly, we have implemented various classification standards for autophagy modulators and autophagy-related genes/proteins respectively, which also provides convenient retrieval for users. First, compounds are divided into four categories based on their source: 1) compounds organically synthesized compounds; 2) monomers isolated from natural products; 3) derivatives and metabolites; 4) extracts and compound preparations. Notably, the derivatives included in AMTDB refer to the more complex products derived from the structure of monomers (obtained from natural products) in which hydrogen atoms or groups are replaced by other atoms or groups. For example, quinoline derivatives of tetrahydrocurcumin and zingerone, 3-Acetyl dihydroxy-olide oleanolic derivative, etc. Secondly, compounds are classified as activator and inhibitor according to their effects on autophagy. In this context, the above concept has been redefined, for example, activator means those that induce autophagy, promote autophagy or increase autophagosome/flux either directly or indirectly. Instead, compounds that block, inhibit, and reduce autophagy, a biological process, are defined as inhibitors. Notably, some compounds have different autophagy effects in different diseases. In terms of gene and protein, type I refers to that the gene has been both reported in the literature and included in the autophagy-related database. Type II is only predicted by the database as autophagy-related genes, but there is no literature to support it, which is a potential target for designing autophagy modulators. Gene that do not fall into the above two categories are automatically classified as type III, which are mostly inherent targets of compounds and are later found to regulate autophagy.

### Similarity calculations

Compounds that can exert biological activity to achieve the purpose of treating diseases are mediated by the binding of their molecules to biological macromolecules such as proteins in the body, which requires the support of binding pockets. The more similar the compounds are, the more likely they are to act on the target with the same spatial configuration. It is well known that virtual screening based on molecular similarity is based on the “similarity hypothesis,” that is, compounds with similar structures have similar physical chemistry information and biological activities (Pavadai et al., 2017). Currently, molecular similarity can be evaluated using descriptors at both 2D and 3D levels. 2D descriptors are calculated from 2D molecular graphs or structural fragments, such as topological indices, molecular fingerprints, etc. For example, a fingerprint is a representation of the molecular structure of a binary format, in which the overlap between molecular fingerprints is used to calculate the Structural similarity between molecules (Maggiora et al., 2014). *Tanimoto Coefficient* is widely used in 2D molecular similarity calculation because of its fast and simple calculation speed (Racz et al., 2018).

Since compounds are essentially three-dimensional, the complex spatial conformation contains much richer information. Therefore, similarity assessment by 3D shape is also allowed, depending on the degree of molecular shape overlap. The *SHAFTS* algorithm uses the descriptor to calculate the shape score and the feature score respectively, and the addition of the two is the final evaluation standard (Gong et al., 2013). Table 1 **.**

**TABLE 1:** Similarity calculation formula.

	Formula		References
*Tanimoto Coefficient* ^a^	The formula for continuous variables	The formula for dichotomous variables	Bajusz et al. (2015)
	$S_{A,B}=\frac{\left[\sum_{j=1}^{n}x_{jA}x_{jB}\right]}{\left[\sum_{j=1}^{n}{\left(x_{jA}\right)}^{2}+\sum_{j=1}^{n}{\left(x_{jB}\right)}^{2}-\sum_{j=1}^{n}x_{jA}x_{jB}\right]}$	$S_{A,B}=c/\left[a+b-c\right]$	
*SHAFTS* ^b^	Shape Score	Feature Score	Liu et al. (2011)
	$V_{AB}=\sum_{i\in A}\sum_{j\in B}\int d\;\vec{r}\;\rho_{i}\left(\vec{r}\right)\;\rho_{j}\left(\vec{r}\right)=\sum_{i\in A}\sum_{j\in B}\;p_{i}p_{j\;}exp\left(-\frac{\gamma_{i}\gamma_{j}}{\gamma_{i}+\gamma_{j}}\right){\left(\frac{\pi }{\gamma_{i}+\gamma_{j}}\right)}^{\frac{3}{2}}$ $ShapeScore=\frac{V_{AB}}{\sqrt{V_{A}V_{B}}}$	$F_{AB}=\sum_{f\in F}\sum_{i\in A}\sum_{j\in B}exp\left[-2.5{\left(\frac{d_{ij}}{R_{f}}\right)}^{2}\right]$ $\mathrm{Feature Score=}\frac{F_{\mathrm{AB}}}{\sqrt{F_{A}F_{B}}}$	
	Hybrid Score=Shape Score+ω⋅Feature Score		

a $x_{jA}$ means the *j*-th feature of molecule A . *a* is the number of *on* bits in molecule A , *b* is number of *on* bits in molecule B , while *c* is the number of bits that are *on* in both molecules.

b i  and  j  represent atoms A and B respectively; $d_{ij}$ :the interatomic distance between atom  i   and j ; γ : is the width of a Gaussian which is relevant with atomic van der Waals radii. where i and j run over the feature points with the same type f in A and B , respectively, $d_{ij}$ is the distance between point i and j , and $R_{f}$ is the overlap tolerance with a default value of 0.8 Å. ω  default weighting factor of 1.0

### Website design

We have a detailed plan for long-term maintenance and changes to the database. AMTDB is deployed on a Linux server and integrates the collated valid data into a MySQL database. We adopted a lightweight Web server (Ngnix version 1.8.1) that alleviates memory pressure and supports responses up to 50,000 concurrent connections (Ma and Chi, 2022). In addition, the Django (version 3.2.13) framework, written in Python, is used to render the Graphical User Interface. The web front end consists of Bootstrap and Asynchronous JavaScript and XML (AJAX) to asynchronously update data and refresh portions of the page. Google Chrome, Safari, and Firefox all have friendly access to the AMTDB (https://amtdb.vercel.app/).

## Results

### Contents of a comprehensive database of autophagic modulators

The AMTDB database, a comprehensive database of autophagy regulators and autophagy-related genes, is dedicated to elucidating the important role of autophagy as a biological process in tumors.

**FIGURE 2:**
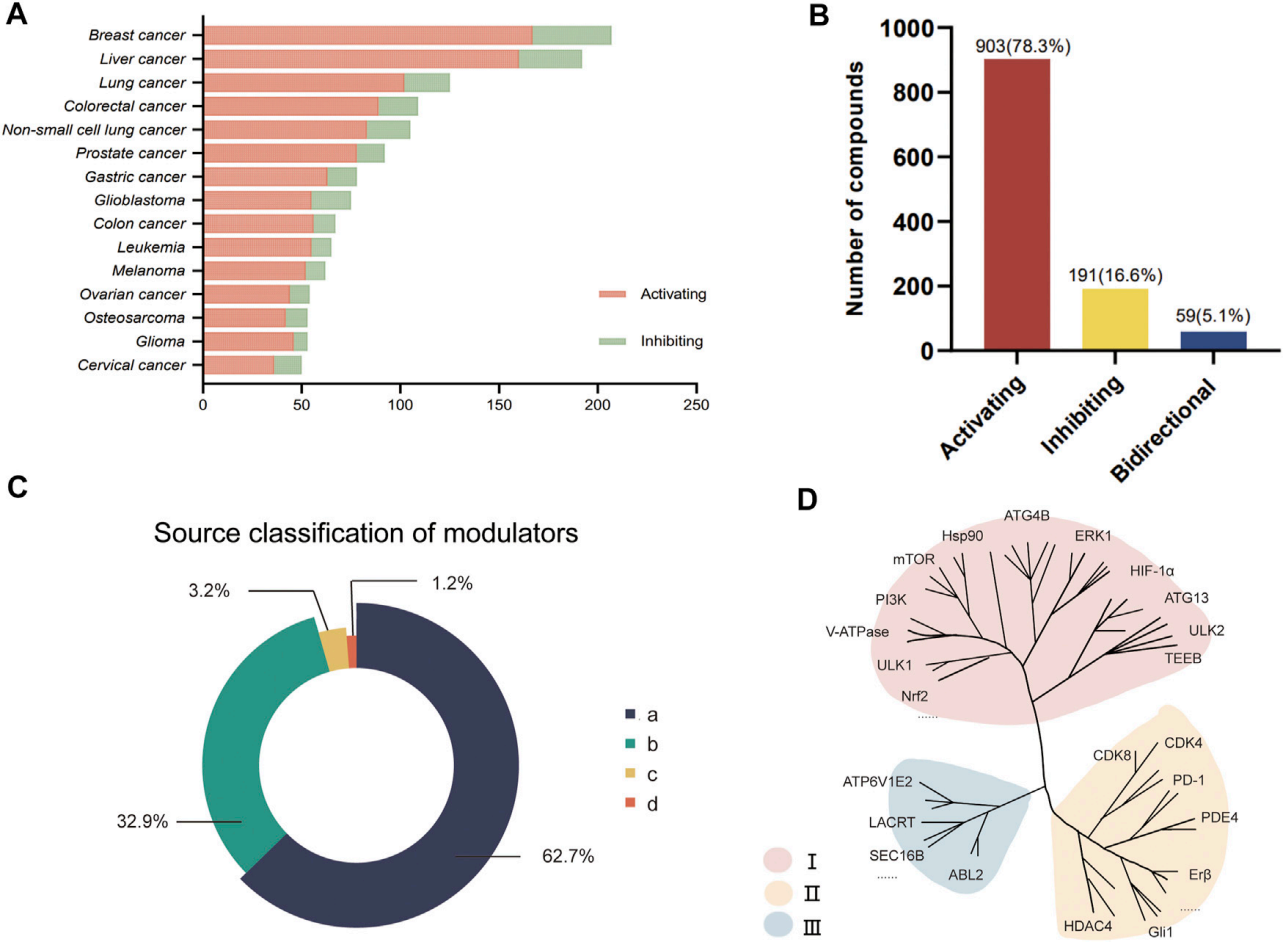
Data visualizations. **(A)** Stacking bars show the function of autophagy modulators in different tumors, with only the top 15 tumor types listed here; **(B)** Functional distribution diagram of autophagy regulator; **(C)** Classification of autophagy modulators in origin attributes; **(D)** Data distribution of autophagy targets contained in AMTDB.

Through text mining of 11,523 published references, we finally obtained 1,153 autophagy regulators, 860 targets, 153 diseases, and involved 2,046 molecular mechanisms. Notably, autophagy-related genes in the GO database and HADb database were included in the AMTDB database as potential targets. The database provides the UniProt ID, organism, protein name, and length information of the autophagy target. To manage such data more clearly, classification criteria are strictly enforced. Targets proved by both the database and literature materials were identified as class I (70.5%); those that only existed in the database and were not supported by reference were class II (5.0%). Class III (24.5%) refers to some targets that have been reported in the literature but not included in the autophagy database, which are often the original targets of compounds but have been found to affect autophagy in later studies Figure 2.

According to statistics, 903 compounds induce autophagy as autophagy activators, 191 are autophagy inhibitors, and 59 compounds have different effects on autophagy in different tumors. Subsequently, during the data collection process, another classification was also placed in the AMTDB database. 1,153 autophagy modulators are meticulously divided into four categories according to their sources, 1) organic synthetic compounds (723, 62.7%); 2) monomer compounds derived from natural products (379, 32.9%); 3) derivatives and *in vivo* metabolites (37, 3.2%); 4) extraction Substances and compound preparations (14, 1.2%). In the autophagy modulators section, the basic information of the compounds is comprehensively compiled, including IUPAC Name, SMILES, CAS, target, disease, and molecular mechanism. Here, the disease refers specifically to tumors, especially cancer, such as breast cancer, ovarian cancer, oral squamous cell carcinoma, osteosarcoma, etc. are included. As an oral potent AMPK activator, ASP4132 was found in a recent non-small cell lung cancer study to induce AMPK phosphorylation and increase AMPK activity, thereby affecting the downstream mTORC1 inhibitory events, thereby inducing autophagy (Xia et al., 2021). It is worth noting that duplicate data has been excluded to avoid invalid and lengthy data from corrupting the reliability of the database. For example, Everolimus and RAD001 are the same compound but appear in different forms in different papers. To avoid data confusion, we unify the compound names (Avniel-Polak et al., 2018; Haas et al., 2019). Furthermore, 3-MA is an effective autophagy inhibitor, which has been reported in many references of breast cancer, but its molecular mechanism is widely used as a class III PI3K inhibitor to inhibit autophagy. Therefore, we give priority to retaining high-level articles and put others in the Ref. column for reference. The above information can be displayed in the search and browse section.

Furthermore, the prediction function was adopted by AMTDB as an online tool for autophagy small molecule target prediction. Target prediction is essentially based on the principle of molecular similarity and is structured in two calculation modes, *Tanimoto Coefficient,* ands *SHAFTS*, aiming to afford users with potential target information.

### Database usage instructions

**FIGURE 3:**
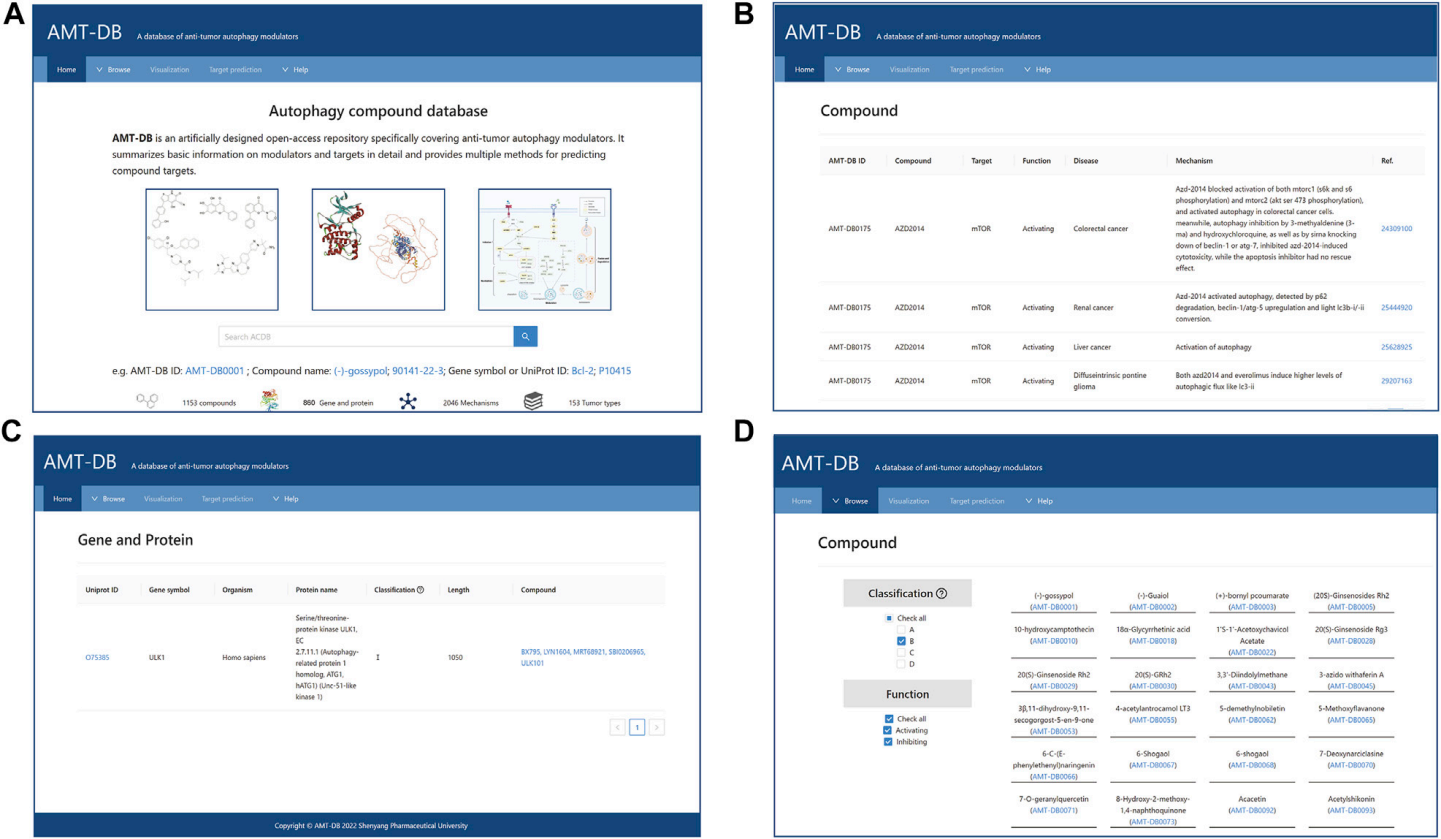
Database in web interface. **(A)** Home page. On this page, users can perform free text retrieval, compound and target information browsing, target prediction, and information submission; **(B)** Quick search. The web page presents information on the pharmacological mechanisms of compounds. **(C)** Gene and protein web interface; **(D)** Browsing interface. Quick access to all compound or gene information. In addition, purpose screening can be performed.

AMTDB is composed of multiple functional sections such as retrieval, browse, predict, and upload, and has been published on webpage https://amtdb.vercel.app/, which implements various needs of users Figure 3A.

#### Retrieval

Users can quickly search for compounds or targets in the toolbar of the Home interface. AMTDB ID, CAS, and compound name were used to query autophagy regulator information. For example, by entering AZD 2014 (1009298-59-2), the web page provides chemical information for the compound, including IUPAC Name, CAS, SMILES, and structure. Click on the AMT-ID on the report page, and detailed pharmacological information will appear, as shown in Figure 3B. It is worth mentioning that AMTDB ID is a custom numbering method of the database, which can be found in the sub-pages of retrieval and browsing, to facilitate the management of background data. Similarly, by entering a Gene symbol or UniProt ID, users can obtain gene/protein information and learn about compounds that modulate this target (Figure 3C).

#### Browse

Under the browse module, the webpage lists all compounds or autophagy-related genes/proteins respectively. Users can categorize compounds or targets using the interactive filters provided on the left. For example, by selecting “b" on the compound browsing page, we obtain 379 monomeric compounds derived from natural products. Additionally, more detailed information can be achieved by hyperlinks (Figure 3D).

#### Visualization

To reduce the user’s learning time cost, we have added a visualization function in AMTDB. The statistical results of tumor type, compound classification, and target classification are visually displayed, and users can click on the results to obtain the desired data.

#### Target prediction

**FIGURE 4:**
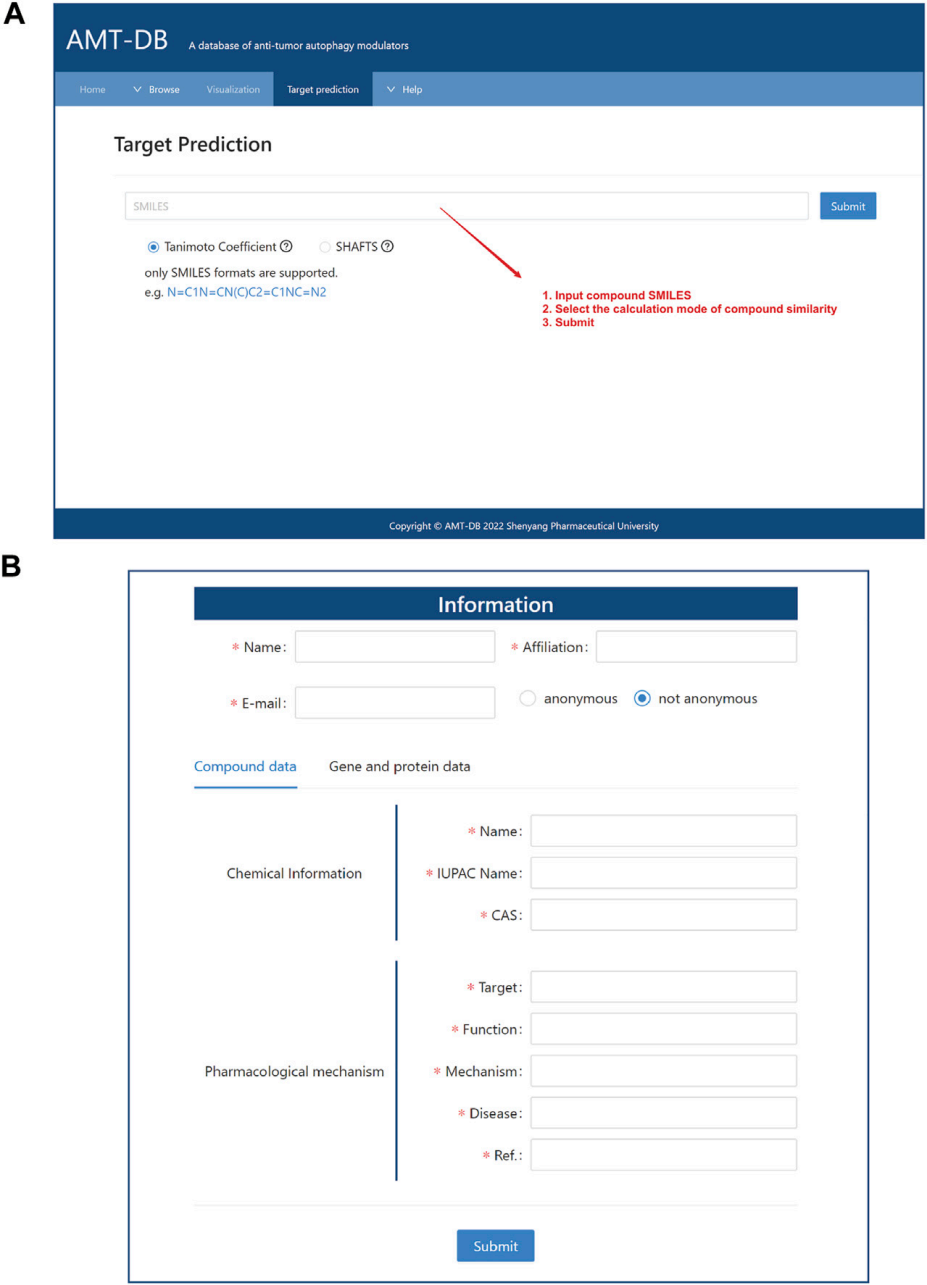
Web interface. **(A)** Target prediction; **(B)** The submit interface.

Under the sub-page of this section, two molecular similarity calculation methods are provided. As shown in Figure 4A, users can input the SMILES of small molecules, and the database will score the submitted molecules by *Tanimoto Coefficient* or *SHAFTS* and compare the similarity with the molecules in the library to predict potential targets. The calculation formulas of the two methods are shown in Table 1. Finally, users get target information and similarity scores from web pages. Of course, compounds of the same target (listed in AMTDB) are also provided to the user for reference. The two prediction methods are different. *Tanimoto Coefficient* predicts at the 2D level, which is fast and widely used, while the *SHAFTS* algorithm calculates the similarity of spatial shapes. Although the latter is theoretically more accurate, the amount of computation is relatively large.

#### Upload

The AMTDB database is based on massive text mining. We must admit that after the data collection is completed, some newly published documents have not been included yet. Herein, we provide an upload function and welcome users to upload autophagy compounds and targets that are not in the database. Undoubtedly, we will update the data from time to time, aiming to provide users with a reliable and practical autophagy-related database (Figure 4B).

### Comparisons with other databases

After nearly a decade of efforts, autophagy has achieved considerable development, and increasing studies have prompted the generation of autophagy-related databases. At present, there are already some databases such as ACDB, and ATdb are opened. However, AMTDB focuses on both autophagy regulators and targets and is peculiar due to added visualization and target prediction capabilities. As shown in Table 2, HADb mainly includes human genes and proteins directly or indirectly involved in autophagy, while ncRDeathDB attempts to decipher the relationship between non-coding RNAs (ncRNAs) and programmed cell death (Moussay et al., 2011; Wu et al., 2015). ATdb, a powerful online database, provides a comprehensive introduction to autophagy and tumors, including 25 types of tumors, survival analysis, and DNA methylation (Chen et al., 2020). Nevertheless, the ATdb database lacks compound information and instead closely links autophagy to the clinic. Compared to compound databases (ACDB, HAMdb, and AutophagySMDB), the AMTDB database compiles compounds and autophagy targets and condenses all data. First of all, ncRNAs and endogenous regulators, such as angiotensin, heparin, and lipopolysaccharide, are not allowed (Deng et al., 2018; Wang et al., 2018; Nanduri et al., 2019). AMTDB contains more autophagic compounds than other databases such as ACDB, and the validity of the data is also an important reference rule for database data quality scores. Third, autophagy-related targets were provided, and confidence intervals were delineated based on target attributes and literature evidences, with a view to presenting a more reliable online resource. Of note, in autophagy-related database, we provide target prediction and visualization function innovatively, which have greatly enriched the database, providing clues for the development of antitumor drugs.

**TABLE 2:** Comparisons of autophagy-related databases.

	Year	Topic	Compound	Target	Disease	Mechanism	Target prediction	Visualization	References
HADb	2011	Gene	−	234	−	+	−	−	−
ncRDeathDB	2015	ncRNA	−	+	−	+	−	−	+
ACDB	2017	Compound	357	+	+	+	−	−	+
THANATOS	2018	Regulation	−	4237	−	−	−	−	+
HAMdb	2018	Compound	841	796	+	+	−	−	+
AutophagySMDB	2019	Small molecules	∼10000	71	+	+	−	−	+
ATdb	2019	Tumor	−	+	+	+	−	+	+
AMTDB	2022	Compound/target	1153	860	153	2046	+	+	+

## Discussion

Autophagy, as we know, is an important biological function of maintaining the intracellular environment, which is strongly induced when lacking of oxygen, stress, and nutrients. Because of its key role in tumor cells, it has caused active intervene to become effective strategies for treating tumors, especially HCQ effective applications in tumor clinical experiments (Karasic et al., 2019). However, autophagy plays a complex and subtle role in tumors, showing both protective and killing functions of cancer cells. For example, casein kinase 1 alpha 1 activates PTEN/AKT/FOXO3A/ATG7 axis-mediated autophagy, inhibiting the growth of non-small cell lung cancer (Cai et al., 2018). Conversely, another study reported that inhibiting autophagy attenuated pancreatic stellate cell activation, thereby preventing pancreatic cancer cell growth and metastasis (Endo et al., 2017). Thus, both autophagy activators and autophagy inhibitors are potential therapeutic agents.

Considering the essential role of autophagy in tumors and the great therapeutic prospects of autophagy modulators, it is particularly important to construct a comprehensive database of compounds, autophagy targets, and molecular mechanisms. AMTDB includes 1,153 autophagy regulators, 153 diseases, 860 targets. Although AMTDB as an online resource related to autophagy is not proposed for the first time, its uniqueness is of great significance for the development of anti-tumor drugs. First, in order to provide users with a more practical and comprehensive database, the AMTDB database adds target prediction function and visualization, which facilitates the discovery of compound targets and provides a convincing path for the discovery of anti-tumor drugs. Secondly, it takes a bridge between autophagy regulators, autophagy-related genes, and tumors, highlights the complex mechanism relationship between the three. Third, AMTDB systematically classifies compounds and autophagy targets, effectively distinguishing natural products, organic synthetic compounds, extracts, and derivatives, which greatly ameliorates the convenience and purpose of retrieval. Finally, emerging articles has resulted in the accumulation of information on autophagy regulators and targets, which requires a new database to be aggregated and updated. Indubitably, AMTDB still has some shortcomings, such as the role of ncRNA in manipulating autophagy cannot be underestimated (Zhao et al., 2020; Zhao et al., 2022). Therefore, we will continue to enrich the data information in later new versions. Although the existing data has been repeatedly checked and randomly verified, the efficiency and accuracy need to be further improved compared with rigorous algorithmic mining.

In conclusion, AMTDB, a comprehensive database dedicated to aggregating autophagic modulators and relevant targets, has been successfully established. In the future, the data in the AMTDB will be further expanded with the explosive growth of autophagy-related investigations, which is a foreseeable development trend. More advanced bioinformatics and artificial intelligence algorithms will be introduced by AMTDB, which would provide an unprecedented opportunity for the discovery of tumor drugs.

## Data Availability

The original contributions presented in the study are included in the article/supplementary material, further inquiries can be directed to the corresponding authors.

## Abbreviations

3-MA, 3-methyladenine; AMTDB, a comprehensive database of autophagy regulators; AMPK, adenosine 5-monophosphate activated protein kinase; AJAX, asynchronous JavaScript and XML; CQ, chloroquine; GO, gene ontology; HCQ, hydroxychloroquine; HIFs, hypoxia-inducible factors; IUPAC, international union of pure and applied chemistry; LC3, microtubule-associated protein 1 light chain 3; ncRNAs, non-coding RNAs; ROS, reactive oxygen species; TFEB, transcription factor EB; ULK1, Unc-51-like kinase 1.
